# Supplementary material for: A New Diterpene with Cytotoxic Potential Against Human Tumor Cells
Source: Molecules. 2025 Dec 2;30(23):4629. doi: 10.3390/molecules30234629 (PMC12693240; doi:10.3390/molecules30234629)
Supplement: Supplementary file 1 [file molecules-30-04629-s001.zip › molecules-3979953-supplementary.pdf]

Supplementary Materials

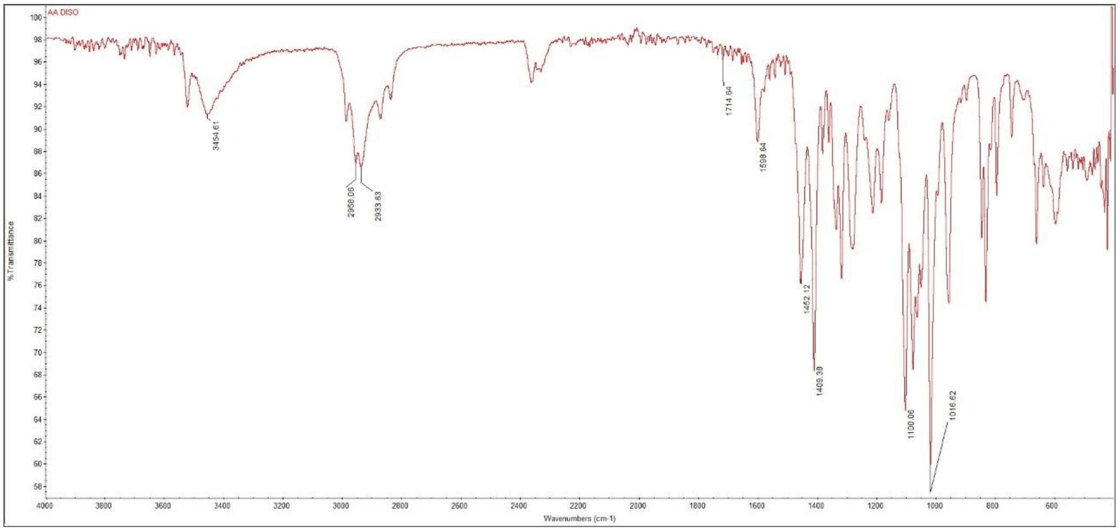

Figure S1. FT-IR spectrum of compound **2** (biisoespintanol).

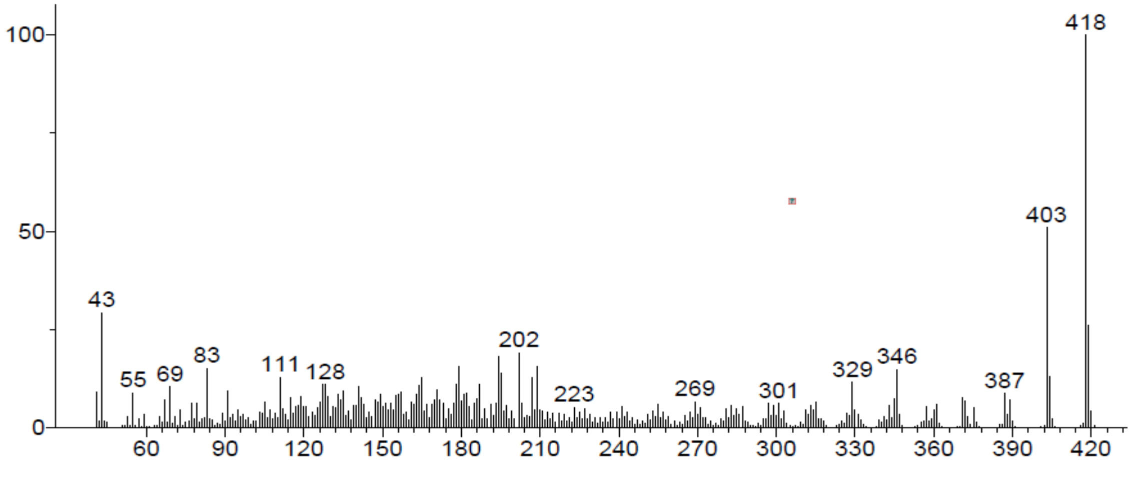

Figure S2. MS spectrum of compound **2** (biisoespintanol), 2mg/mL dichloromethane.

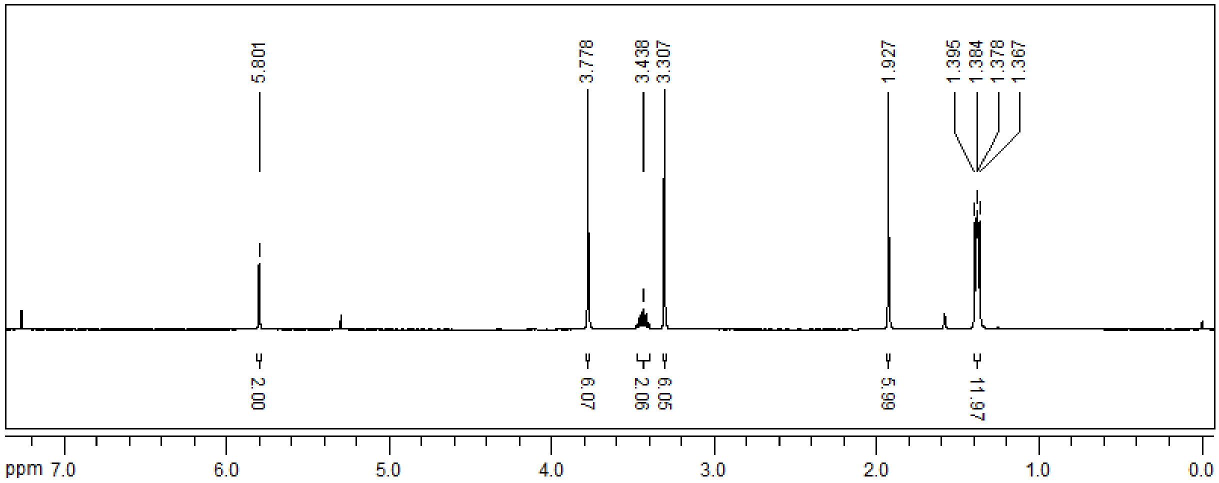

Figure S3. ^{1}H-NMR spectrum (400 MHz, $CDCl_3$) of compound **2** (biisoespintanol).

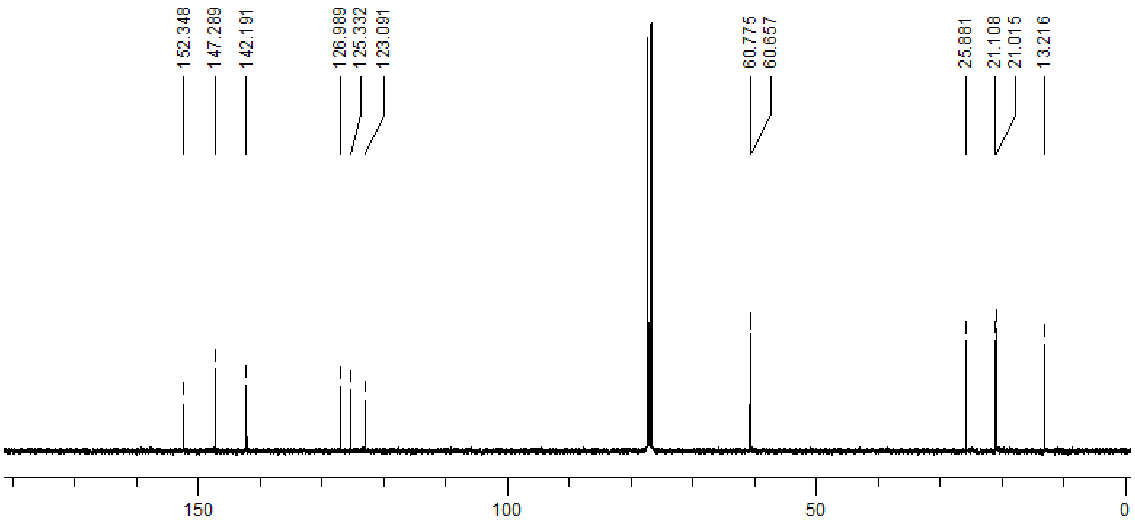

Figure S4. ^{13}C-NMR spectrum (100 MHz, $CDCl_3$) of compound **2** (biisoespintanol).

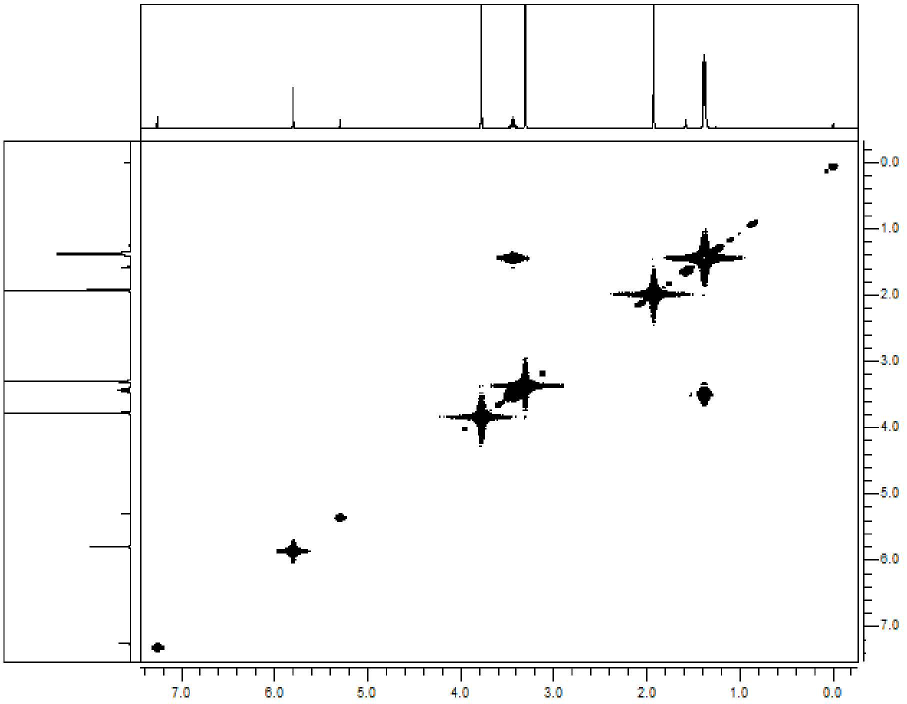

Figure S5. COSY NMR spectrum (400 MHz, $CDCl_3$) of compound **2** (biisoespintanol).

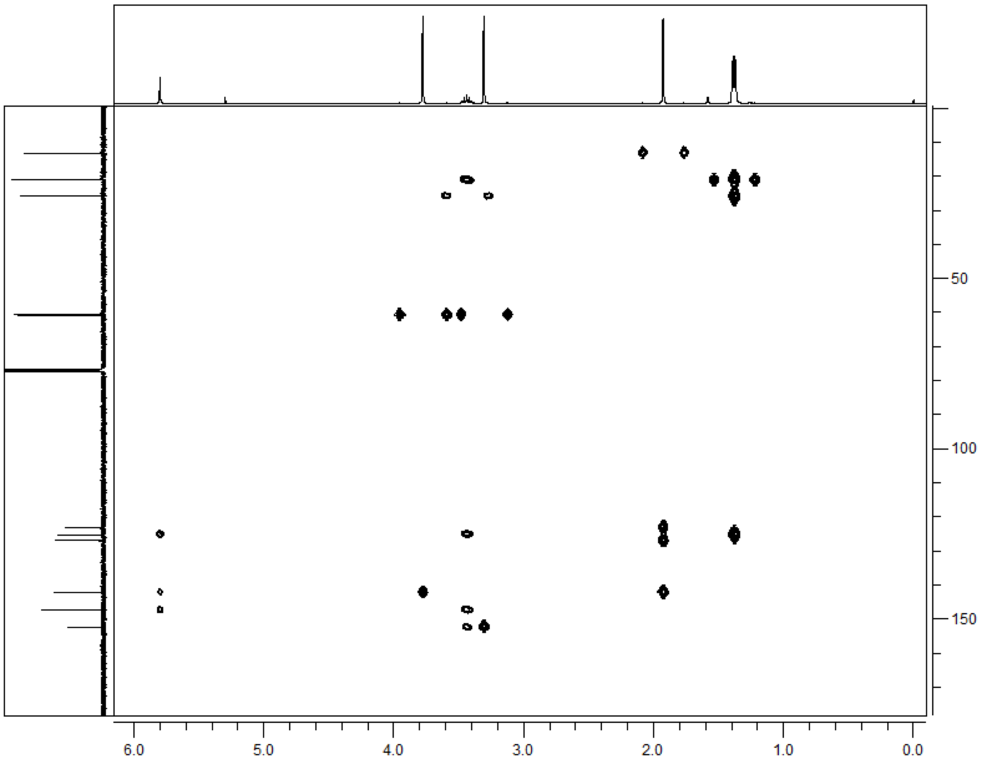

Figure S6. HMBC NMR spectrum (400 MHz, $CDCl_3$) of compound **2** (biisoespintanol).

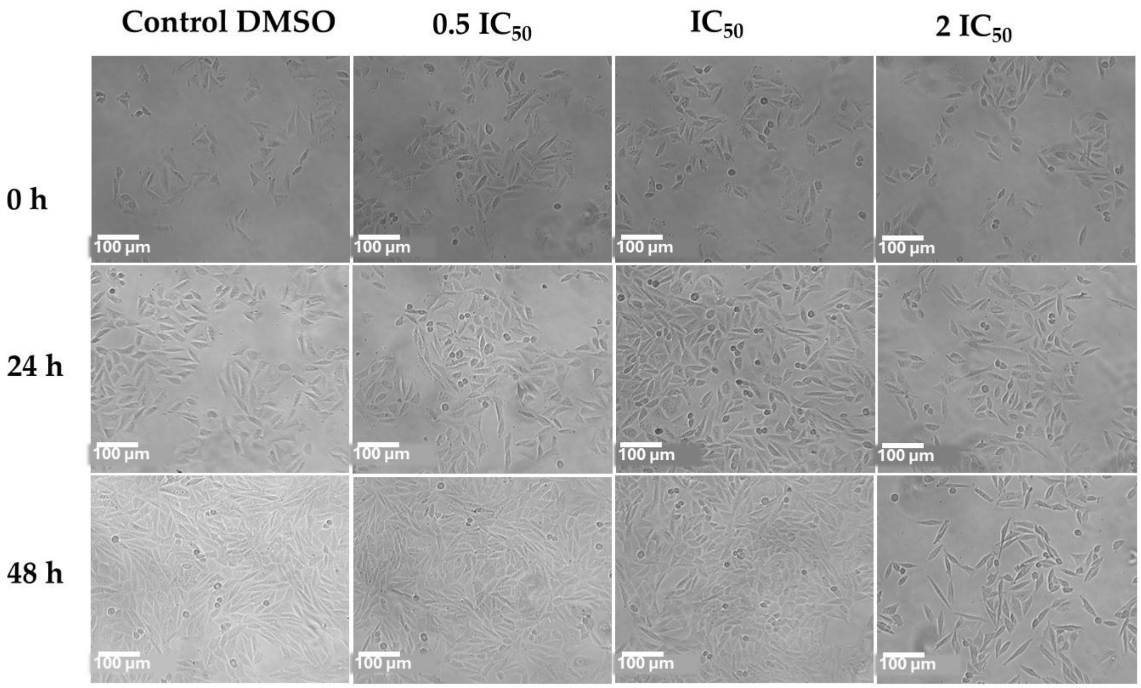

Figure S7. Brightfield microscopy of the morphological study in non-tumor lung cells (MRC-5) after treatment with compound **2** (0.5 IC50, IC50 and 2 IC50) at 0, 24 and 48 h; 10X magnification. No significant morphological changes were observed after treatments with IC50 and 2 IC50 at 24 and 48 h. The cells were photographed with the CELENA® S digital imaging system (Logos Biosystems).

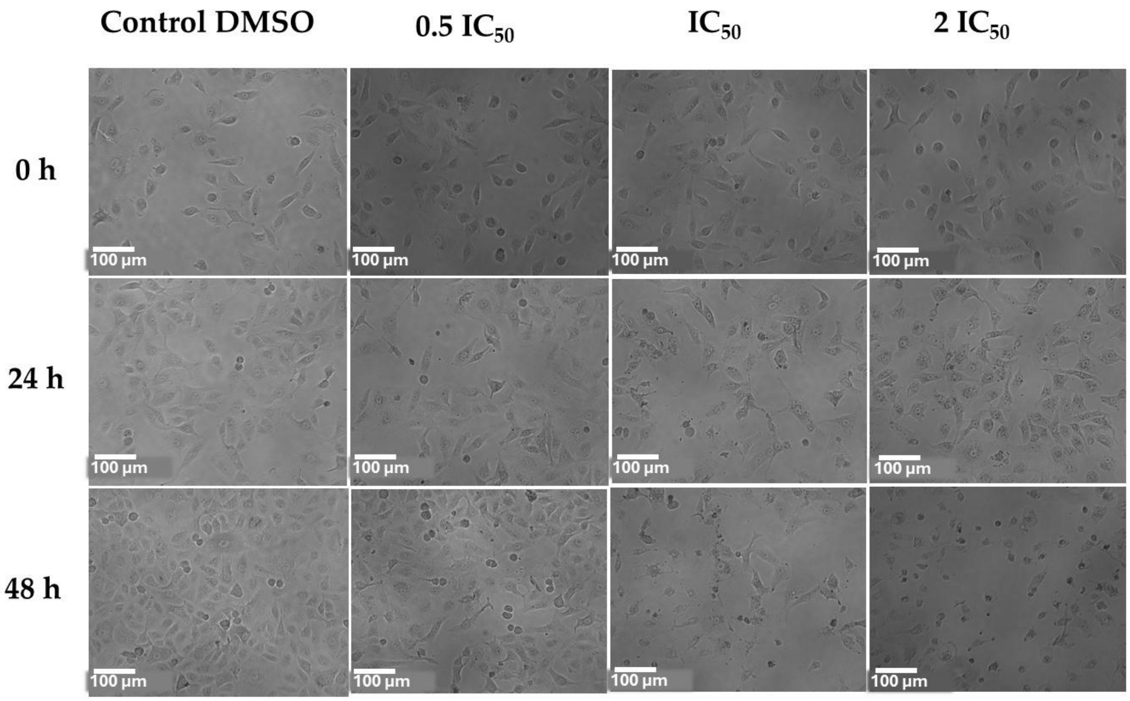

Figure S8. Brightfield microscopy of the morphological study in lung tumor cells (A549) after treatment with compound **2** (0.5 IC50, IC50 and 2 IC50) at 0, 24 and 48 h; 10X magnification. Significant morphological changes were observed with treatments with IC50 and 2 IC50 at 24 and 48 h. The cells were photographed with the CELENA® S digital imaging system (Logos Biosystems).
